# Convergent community structure of algal–bacterial consortia and its effects on advanced wastewater treatment and biomass production

**DOI:** 10.1038/s41598-021-00517-x

**Published:** 2021-10-26

**Authors:** Feng Qi, Yantian Jia, Ruimin Mu, Guixia Ma, Qingyang Guo, Qianya Meng, Gejiang Yu, Jun Xie

**Affiliations:** 1 School of Municipal and Environmental Engineering, Shandong Jianzhu University, Jinan, 250101 China; 2 Shandong Provincial Eco-Environment Monitoring Center, Jinan, China

**Keywords:** Microbial communities, Environmental microbiology, Pollution remediation

## Abstract

Microalgal-bacterial consortium is an effective way to meet increasingly stringent standards in wastewater treatment. However, the mechanism of wastewater removal effect has not been properly explained in community structure by phycosphere. And little is known about that the concept of macroecology was introduced into phycosphere to explain the phenomenon. In the study, the algal–bacterial consortia with different ratios of algae and sludge were cultured in same aerobic wastewater within 48 h in photobioreactors (PSBRs). Community structure at start and end was texted by metagenomic analysis. Bray–Curtis similarities analysis based on microbial community showed that there was obvious convergent succession in all consortia, which is well known as “convergence” in macroecology. The result showed that Bray–Curtis similarities at End (overall above 0.88) were higher than these at Start (almost less than 0.66). In terms of community structure, the consortium with 5:1 ratio at Start are the more similar with the consortia at End by which the maximum removal of total dissolved nitrogen (TDN, 73.69%), total dissolved phosphorus (TDP, 94.40%) and NH_3_-N (93.26%) in wastewater treatment process and biomass production (98.2%) higher than other consortia, according with climax community in macroecology with the highest resource utilization than other communities. Therefore, the macroecology can be introduced into phycosphere to explain the consortium for advanced wastewater treatment and optimization community structure. And the study revealed a novel insight into treatment effect and community structure of algal–bacterial consortia for advanced wastewater treatment, a new idea for to shortening the culture time of consortium and optimize predicting their ecological community structure and predicting ecological community.

## Introduction

Most suspended solids and organic pollutants can be removed by primary and secondary treatment of wastewater. Then the removal effect of nitrogen and phosphorus needs additional tertiary treatment to realize nitrogen and phosphorus removal, which increases the cost of wastewater treatment. Wastewater treatment needs additional energy and steps to realize nitrogen and phosphorus removal, which increases the cost of wastewater treatment^1^. The cost of aeration in the activated sludge process accounts for 45–75% of the cost of wastewater treatment^2^. In this process, the emissions of carbon dioxide and nitrogen oxide also caused environmental pollution^3–5^. In addition, excess sludge is also a “pollution transfer”^2^.

Microalgae has overcome the limitations of traditional wastewater treatment methods^6,7^. In recent years, the research on the absorption and transformation of nitrogen and phosphorus by the symbiotic system of algae and fine bacteria has aroused widespread concern^8^. The algal–bacterial consortium system has been favored by scholars due to its unique characteristics, such as reduced power consumption and biomass refinability^8^. Its principle is to use the relationship between algal bacteria and community structure to promote the effect of removing organic pollutants by heterotrophic bacteria and N, P by algae from wastewater^4,9^. Aeration provides excess oxygen to COD-degrading bacteria. Obviously, oxygen provided by algae photosynthesis is not useful for bacteria to remove COD. Therefore, bacteria have a dominant position in the community in the group with high COD removal efficiency. For example, the high algae/sludge ratios constitute algal-dominated algal–bacterial consortia which remove the nitrogen and phosphorus by algae absorption^10^. However, when the concentration of activated sludge is higher than that of algae in consortia, it shows better COD removal ability^8^. Due to poor photosynthesis, denitrification depends on nitrifying bacteria and denitrifying bacteria^8^. In addition, hydraulic retention time (HRT) were reduced by highly mixed liquid suspended solids. There is a suitable example that the high rate alga pond (HRAP) the sufficient mixing with mechanical mixing by paddlewheels reduced prominently HRT than stabilization ponds. For example, Solimeno et al. argued that HARP system with the benefit of wastewater treatment effect from alga-bacteria co-culture makes them more attention over stabilization ponds. And they found average removal efficiency of ammonium goes up to 92% in HRAP model. Besides, improving biomass production of algal–bacterial consortium was widely reported, such as operational strategies optimizing (rector optimizing, HRT and additional CO_2_), consortia recirculation and pre-disinfection. However, the competitive relation between algae and bacteria was reflected by the nutrient competition. Therefore, suitable Light intensity and resource concentration are worth considering.

Although some studies involve the influence of algal and bacteria community structure on wastewater treatment, there is no systematic theories to explain these results, and many of them are contradictory. For example, both consortium at the algae-bacteria ratio of 1:3^11^ and 5:1^10^ have not similar N removal effects (89.0% and 91.0% respectively). This is largely due to the lack of an agreed methodology for measuring structural changes in the algal bacteria consortium.

Phycosphere refers to micro-environment, which the algal–bacterial consortia and the surrounding microbiome, ecological factors and nutrients^12^. The environment, which has resources, light, dissolved oxygen, and stable biological relationships suitable for the development, conforms to the relevant characteristics of the climax community in macroecology^13^. Phycosphere is an ecosystem formed by microorganisms. Thus, the theory well-established in macroecologicaly theory can be tried to be introduced into phycosphere^12^. In fact, macroecological theory has been widely applied to the study of microbial community structure and community relations. For example, the phylogenetic composition and community structure of planktonic bacteria communities in eutrophic floc lake and clear water lakes were analyzed by 16S rRNA sequencing^14^. In addition, community structure of activated sludge also verified the mechanism of wastewater treatment^15^. However, there are few studies on the relationship between the phycosphere from the ecological point of view^16^.

Community succession is a non-static process, which refers to the process that the community structure developed into a specific community structure with the passage of time, which is related to environmental factors, resource and community relations^17,18^. In macroecology, it is generally believed that under certain resources and environmental conditions, different biological communities will succeed to a specific climax community, which can maximize the use of resources and get the best biomass production^19^. Whether there is such a succession result in algae microecology is worth studying, which is of great significance to predict the microbial community structure and shorten the incubation time^19^. The objective method to evaluate the similarity of community is to use the statistical index reflecting the difference in community composition^20,21^. For this reason, dozens of similarity and dissimilarity coefficients (indices) have been designed to compare diversity differences among biota. Bray–Curtis Distance^21^ is used to measure the difference of species composition in different areas in ecology, which can calculate the quantitative characteristics of different species composition in biological samples. It has been widely used in the similarity analysis of bacterial community, algae bloom and aquatic community^22^. It is considered to be the best criterion for reflecting the diversity of different algae communities. The advantage is that using the abundance of different species as the input data to get the results of the difference in the structure of different groups of algae. The results are in line with the expectations. Besides, the Bray–Curtis index has been applied into the field of microecology research in recent years^23^, used as an index to analyze the similarity of microbial community and the distribution of functional genes^24^. Therefore, it is a good choice to apply Bray–Curtis index to the phycosphere.

In the research, five different communities constructed by different biomass ratios of algae and bacteria were cultured in the secondary effluent in the same water quality index within 48 h in PSBR. The community structure was sequenced by metagenomics. The purpose of this study was to explore whether the idea of climax community in macroecology and convergence of community succession could be applied to the phycosphere by Bray–Curtis similarity.

## Materials and methods

### Cultures and media

In this study, the culture was mixed with natural algae community and activated sludge in different proportions. The active algae community culture was isolated from Yingxue Lake of Shandong Jianzhu University and cultured in BG11 medium to logarithmic growth period, when the activity of algae was better than other period^25^. Activated sludge was collected from Reclaimed Water Station of Shandong Jianzhu University, and cultured in artificial wastewater.

The mixing ratio of algae and activate sludge (dry weight)^26^ are as follows: 10:1, 5:1, 1:1, 1:5 and 1:10 (as donations from R1 to R5). In the same wastewater environment, different consortia were cultivated under the same process conditions, such as light intensity, aeration rate, temperature and carbon dioxide consumption.

In this study, the artificial wastewater and secondary effluent from the wastewater treatment plant of Shandong Jianzhu University were taken as research objects. The water quality compositions are (mg L^−1^): Chemical oxygen demand (COD) 110–150, Total phosphorus (TDP) 3–5, Total dissolved nitrogen (TDN) 20–40, Ammonia nitrogen (NH_3_-N) 10–20. And the pH was 7.01–7.05. The artificial wastewater is follows (g L^−1^): NaAH 0.51, MgSO4 0.09, CaCl_2_ 0.014, Na_2_HPO_4_ 0.153, Yeast 0.01 and 1 mL trace elements including (g/L): ZnSO_4_·7H_2_O 0.12, H_3_BO_3_ 0.15, CuSO_4_·5H_2_O 0.03, MnCl_2_·4H_2_O 0.12, KI 0.18, Na_2_MoO_4_ 0.06, EDTA 1, FeSO_4_·7H_2_O 1.54, CoCl_2_·6H_2_O 1.5.

### Experimental setup

**Figure 1:**
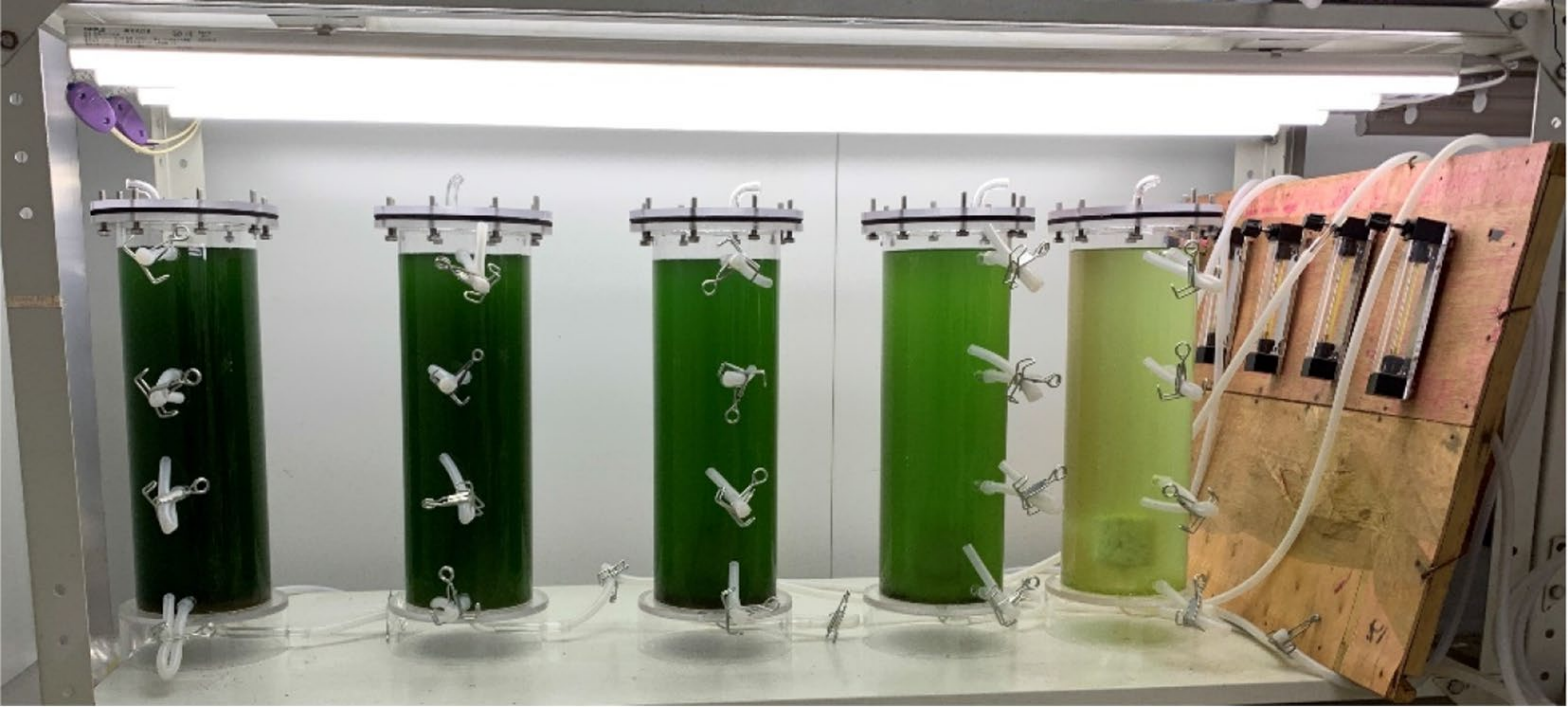
PSBR with Consortia with algae/sludge 10:1, 5:1, 1:1, 1:5 and 1:10 (donated as R1 to R5).

The biomass concentration (dry weight) of algae and bacteria community was 500 mg L^−1^. The dry weight process was: The samples were filtered out of liquid and dried at 105 °C to constant weight. The working volume of the reactor is 3 L, which is made of transparent organic glass. The reactor has a height of 300 mm, the inner diameter of 120 mm, and the wall thickness of 5 mm. The bottom of the reactor is provided with an air inlet which is connected with the aeration head. The top of the reactor is respectively provided with an air outlet and a spare feed inlet. The photobioreactor is shown in Fig. 1.

The experiments were conducted with mature algal–bacterial consortia in 5 uniform photobioreactors. The initial biomass concentration was 500 mg L^−1^; The temperature was 24 degrees; The 95:5 airflow-CO_2_ (v/v) gas mixture into the bottom of the PSBRs with a speed of 0.6 L min^−1^ for each column to agitate the algal broth as well as supply carbon from air. The rubber-pipes were connected with glass connector to convey to the aeration head. The purity of CO_2_ was 99%. The average illuminance was 4000 Lux. The period of batch operation was set at 48 h.

### Analytical methods

In each cycle, samples were collected at intervals of 8 h, and then centrifuged at 4000 r/min for 10 min. The supernatants of samples filtered through 0.45 mm filter for further analysis, and were used for detecting Chemical oxygen demand (COD) determined by Dichromate method (HJ 828-2017), Total dissolved phosphorus (TDP) determined by Ammonium molybdate spectrophotometric method (GB 11893-89), Total dissolved nitrogen (TDN) determined by Alkaline potassium persulfate digestion UV spectrophotometric method (HJ 636-2012) and Ammonia nitrogen (NH_3_-N) determined by Nessler’s reagent spectrophotometry (HJ 535-2009). The standard of methods were determined according to the national standards (Chinese state standard monitoring method)^27^.

The pH was determined to use a PHS-3B pH meter. The biomass concentration was estimated by dry weight (DW) measurement^26^. Each sample was set in triplicate to eliminate unexpected errors.

### Microbial structure analysis

Microbial samples were collected at the start-up stage (Start, the 0 h) and the end of operation stage (End, the 48th h), and then stored at − 80 °C until the genomic DNA was extracted. According to manufacturer’s agreement, microbial DNA was extracted by using the Tiangen DP329 DNA Kit (Tiangen Biotech, Beijing, China). The concentrations and purity of DNA samples were detected by 1% agarose gel electrophoresis. The extracted DNA were amplified by Polymerase Chain Reaction (PCR) (98 °C pre-degeneration for 1 min, followed by 30 cycles at 98 °C for 10 s, 50℃ for 30 s, 72 °C for 30 s and a final extension 72 °C for 5 min). For metagenomics analysis, Illumina Truseq DNA PCR-Free Library Preparation Kit was used to construct the Library^28^. After Qubit quantification and Library test, Novaseq 6000 was used for online sequencing.

### Similarity analysis of community structure

The Bray–Curtis similarity was shown via Eq. (1).

1 $${D}_{Bray-Curtis}=1-2\frac{\sum min({S}_{A, i},{S}_{B,i})}{\sum {S}_{A,i}+\sum {S}_{B,i}}$$

where ${S}_{A, i},{ S}_{B,i}$, are counts of *i* species in community A and community B, respectively. The range from 0 to 1. The closer the value is to 1, the more similar the two communities become. On the contrary, the closer to 0, the greater the difference between the two communities. Analysis of similarities (ANOSIM), based on Bray–Curtis coefficient, may visualize the coefficients and show its significance. The R value was shown via Eq. (2).

2 $$R=\frac{{r}_{b}-{r}_{w}}{\frac{1}{4}\left[n\left(n-1\right)\right]}$$

where ${r}_{b},{ r}_{w}$, are counts of mean rank of between and within group dissimilarities, respectively. The R in the range of − 1 to 1. When R $>0$, it is significant in group. When R $<0$, the group is meaningless.

The Bray–Curtis coefficient and ANOSIM are conducted by PAST 4.0 (http://folk.uio.no/ohammer).

## Results

### Wastewater remediation

**Figure 2:**
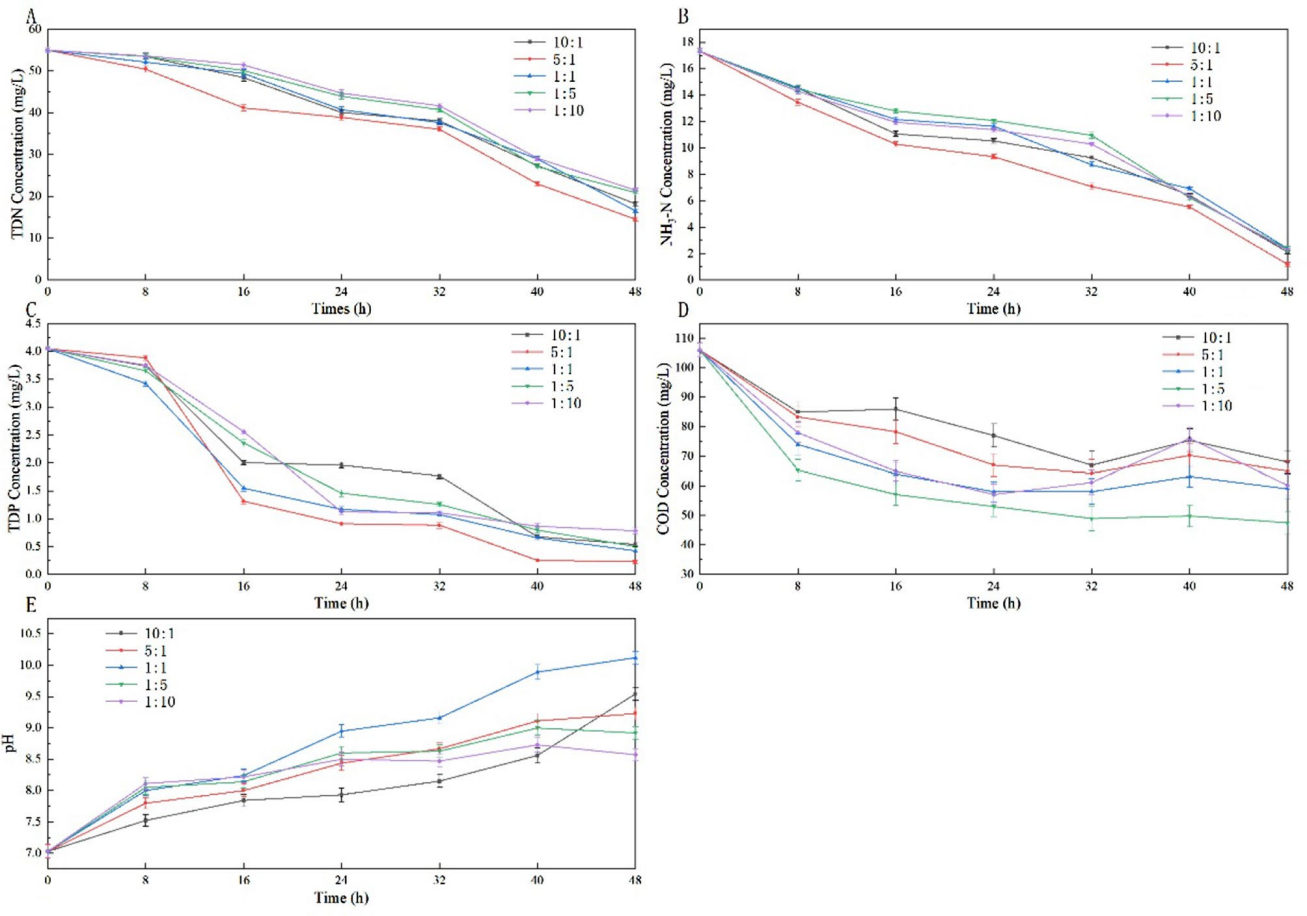
Concentration of water quality compositions in consortia. Consortia with algae/sludge 10:1, 5:1, 1:1, 1:5 and 1:10 (donated as R1 to R5). Nutrient and COD in different ratio of algal–bacterial consortia. And the pH in water. Ammonia nitrogen, total nitrogen, total phosphorus, COD, pH were shown in (**A**), (**B**), (**C**), (**E**) and (**F**) respectively.

Figure 2 showed the nutrient removal of R1 to R5 in PSBR. The TDN removal efficiency in the PSBRs with R1 to R5 were reached to 66.87%, 73.69%, 69.99%, 61.95% and 60.93% respectively after 48 h (Table 1). The PSBR with R2 exhibited the highest and fastest TDN removal, compared with other consortia. In the first 8 h of the reaction, the removal of TDN was not significant. Since then, the removal rate of TDN has been faster than before, especially for R2. Similarly, the NH_3_-N removal efficiency in the PSBRs with R1 to R5 were reached to 87.78%, 93.26%, 86.30%, 86.45% and 87.08% respectively after 48 h (Table 1). The PSBR with R2 exhibited the highest and fastest NH_3_-N removal, compared with other consortia. The TDP removal in these complexes was also investigated. The fastest and highest TDP removal was found in these consortia inoculated with R2 (94.40%), followed by R3, R4, R1 and R5, respectively 89.58%, 87.69%, 86.64%, and 80.63% respectively. Between the 8th and the 16th h, the concentration of TDP decreased significantly with R1 to R5.

**Table 1:** The wastewater removal efficiency and biomassproductivity for consortia with different algae/activated sludge ratios.

Consortium	TDN removal (%)	NH3-N removal (%)	TDP removal (%)	COD removal (%)	Biomass productivity (%)	TDN removal rate (mg L^−1^ d^−1^)	NH3-N removal rate (mg L^−1^ d^−1^)	TDP removal rate (mg L^−1^ d^−1^)	COD removal rate (mg L^−1^ d^−1^)	Biomass productivity rate (× 10^3^ mg L^−1^ d^−1^)
R1	66.87 ± 0.18	87.78 ± 0.09	86.64 ± 0.11	35.75 ± 1.4	41.93 ± 0.3	18.39 ± 0.18	7.60 ± 0.09	1.75 ± 0.01	18.95 ± 1.40	0.18 ± 0.018
R2	73.69 ± 0.26	93.26 ± 0.08	94.40 ± 0.11	38.68 ± 1.1	49.55 ± 0.3	20.26 ± 0.26	8.08 ± 0.08	1.91 ± 0.01	20.50 ± 1.10	0.25 ± 0.018
R3	69.99 ± 0.23	86.30 ± 0.07	89.58 ± 0.11	44.34 ± 1.2	47.31 ± 0.3	19.25 ± 0.23	7.47 ± 0.07	1.81 ± 0.01	23.50 ± 1.20	0.22 ± 0.019
R4	61.95 ± 0.29	86.45 ± 0.06	87.69 ± 0.12	55.19 ± 1.2	40.83 ± 0.28	17.04 ± 0.29	7.49 ± 0.06	1.78 ± 0.01	29.25 ± 1.20	0.17 ± 0.02
R5	60.93 ± 0.25	87.08 ± 0.08	80.63 ± 0.10	43.26% ± 1.2	38.42 ± 0.31	16.76 ± 0.25	7.54 ± 0.08	1.63 ± 0.02	22.93 ± 1.20	0.16 ± 0.02

The mineralization abilities of COD were investigated. The corresponding COD removal efficiencies with R1 to R5 were 35.75%, 38.68%, 44.34%, 55.19% and 43.26% respectively (Table 1). The fastest and highest COD removal efficiency was found in these complexes inoculated with R5. It was obvious that the COD removal efficiencies with R1 and R5, whose difference in the amount of sludge between them. It’s worth noting that the nutrient removal not only is related to the algae concentration, but also high concentration activated sludge affect the removal efficiency at initial stage (N and P removal efficiency R5 $>$ R3 $>$ R4). Within 48 h, the pH value of all the consortia increased. The maximum pH was R3 and minimum was R5. The range of pH was 8.5 to 10.5. After 32 h, the pH value was relatively stable.

### Biomass accumulation

**Figure 3:**
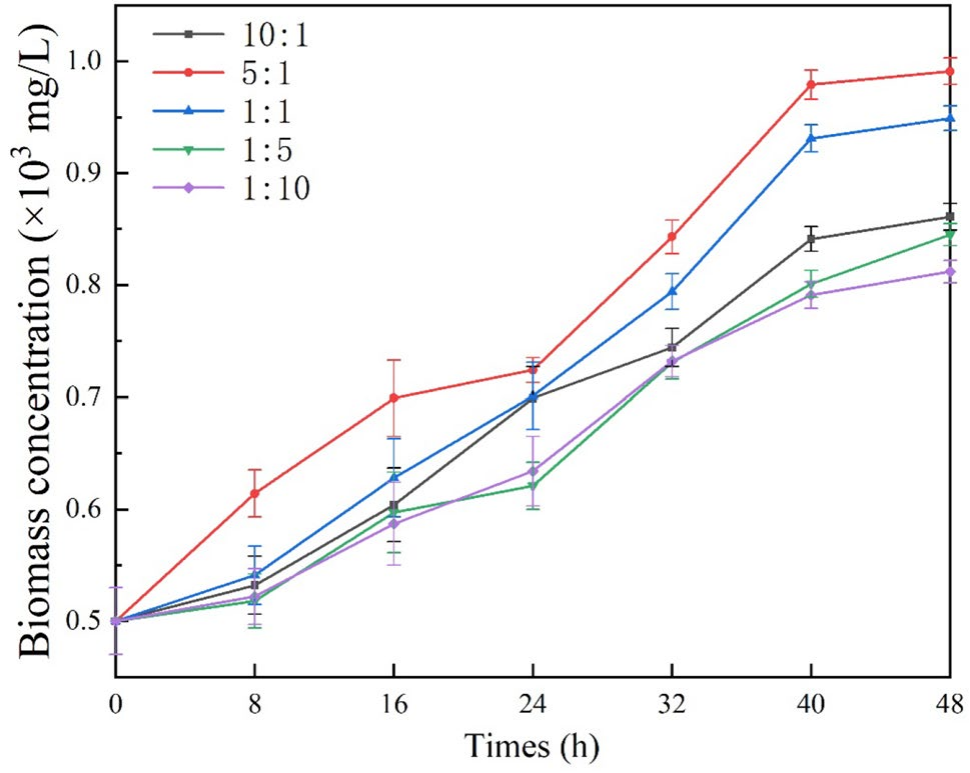
The promotion of the biomass concentration. Consortia with algae/sludge 10:1, 5:1, 1:1, 1:5 and 1:10 (donated as R1 to R5).

Figure 3 shows the increase in the biomass concentration. The biomass concentration of algal–bacterial consortia increased significantly after the absorption of nutrients. The biomassproductivity of R1 to R5 was 72.2%, 98.2%, 89.80%, 69.00% and 62.40% respectively (Table 1). Obviously, the highest growth efficiency of biomass was R2. The biomassproductivity of the complex with high algae concentration were higher than that of the complex with high activated sludge. However, the biomass productivity of R2 (98.2%) was higher than that of R1 (72.2%), and the algae concentration than R2 is higher. On the contrary, the biomassproductivity of the consortium of R4 (69.00%) was higher than R5 (62.40%) in high algae concentration.

The 24th h was an important node. After that, the efficiency of increasing biomass concentration has been improved significantly. The time point for nitrogen removal efficiency improvement was also 24 h. However, TDP removal effect was bigger and faster before the 18th h.

### Convergence of community structure

#### Microbial community structure at Start

**Figure 4:**
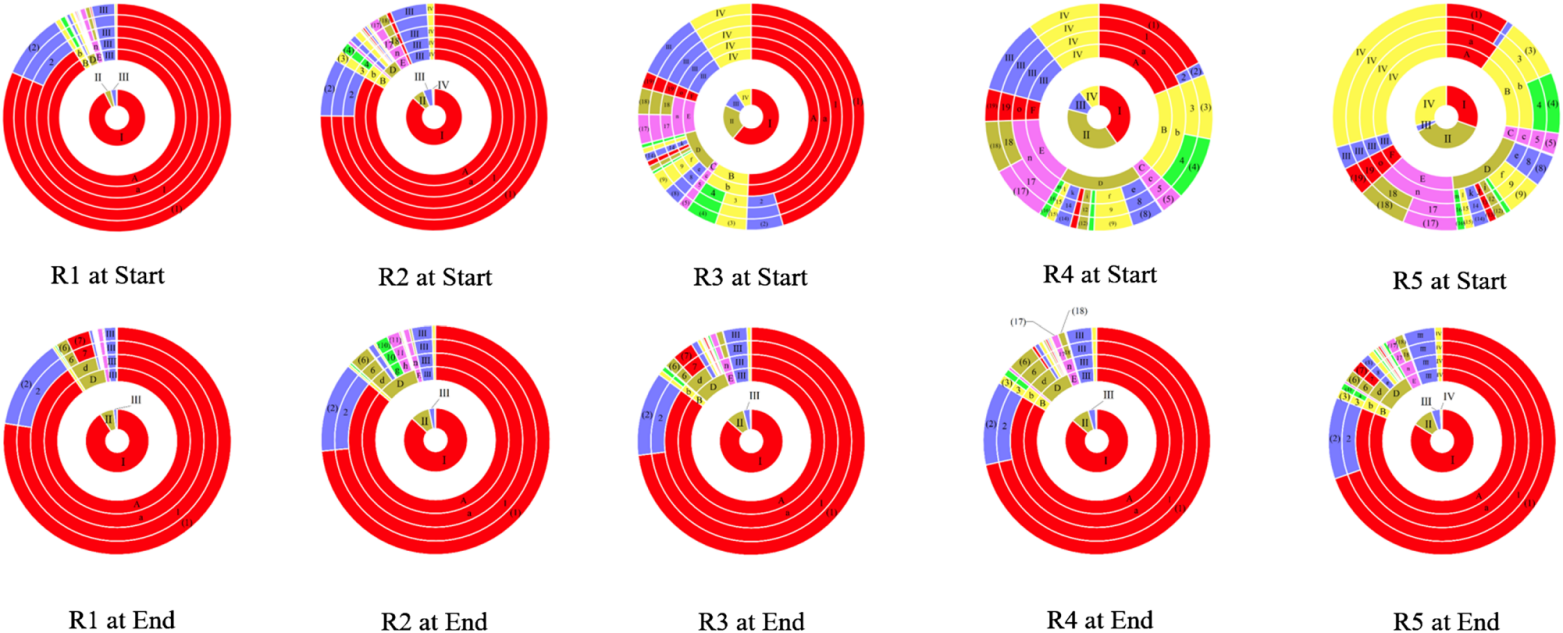
Algal–bacterial Consortia multistage species composition at the Start and End. Consortia with algae/sludge 10:1, 5:1, 1:1, 1:5 and 1:10 (donated as R1 to R5). Start (the operation start-up, the 0 h) and End (the operation end, the 48th h). Autotroph, heterotroph, other of algae, other of bacteria (donated as I To IV). *Chlorophyta, Chlorophyta, Cyanophyta, Chloroflexi, Proteobacteria, Bacteroidetes, Bacteroidetes and Nitrospirae* in Phylum (donated as A to F). *Chlorophyceae, Chlorophyceae, Cyanophyceae, Unclassified_p_Chloroflexi, proteobacteria, Betaproteobacteria, proteobacteria, Gammaproteobacteria, Alphaproteobacteria, Deltaproteobacteria, Unclassified_p_Bacteroidetes, Unclassified_p_Bacteroidetes, Nitrospirales* in Class (donated as a to o). *Chlorococcales, Nostocales, Unclassified_p_Chloroflexi, Pseudomonadales, Burkholderiales, Unclassified_c_Betaproteobacteria, Pseudomonadales, Burkholderiales, Unclassified_c_Gammaproteobacteria, Rhodobacterales, Myxococcales, Unclassified_c_Alphaproteobacteria, Thauera, Unclassified_p_Bacteroidetes, Nitrospirales* in Order (donated as 1 to 15). *Chlorella, Scenedesmus, Anabaena, Spirulina, Unclassified_p_Chloroflexi, Acinetobacter, Unclassified_o_Pseudomonadales, Burkholderiales, Unclassified_c_Betaproteobacteria, Pseudomonas, Comamonas, Unclassified_c_Gammaproteobacteria, Unclassified_o_Rhodobacterales, Unclassified_o_Myxococcales, Unclassified_c_Alphaproteobacteria, Thauera, Unclassified_p_Proteobacteria, Unclassified_p_Bacteroidetes, Nitrospira* donated as (1) to (19).

As shown in Fig. 4 and Table S1, the composition of native algae community is mainly *Chlorella *sp. and *Scenedesmus *sp. by the microscope of R1 to R5. At the same time, some cyanobacteria, such as, *Anabaena *sp. and *Oscillatoria *sp. have also been observed with all consortia.

At Start, the microbial communities were striking separation in R1 to R5, with algae accounting for 92.46%, 86.28%, 59.60%, 32.84% and 27.30% respectively (Table 2); Bacteria community account for 7.541%, 13.72%, 40.40%, 67.12% and 72.70% (Table 2), respectively, which is not similar to each other by Bray–Curtis similarity. As shown in Table 3, the ratio of algae community related to photosynthesis and bacteria community about heterotroph was 12.26:1, 6.29:1, 1.48:1, 1:2.04 and 1:2.66 respectively at Start. The taxonomic classifications of bacterial reads at the phyla level were depicted in Fig. 4. *Proteobacteria*^29^, *Cyanobacteria*^30,31^, *Bacteroidetes*^15^, *Chloroflexi*^32^, *Nitrospirae*^33^ with these consortia were related to nutrient removal. However, *Proteobacteria* remained the dominant species with consortia, microbial function shown in Table 3.

**Table 2:** The abundance of autotrophic micro-organism and heterotrophic microorganism.

Community		Abundance		Ratio
		Autotrophic microorganism (%)	Heterotrophic microorganism (%)	
Start	R1	92.459	7.541	12.26:1
	R2	86.282	13.718	6.29:1
	R3	59.598	40.402	1.48:1
	R4	32.844	67.156	1:2.04
	R5	27.301	72.699	1:2.66
End	R1	90.992	9.008	10.10:1
	R2	86.815	13.185	6.58:1
	R3	86.347	13.653	6.32:1
	R4	85.832	14.168	6.06:1
	R5	83.474	16.526	5.05:1

Consortia with algae/sludge 10:1, 5:1, 1:1, 1:5 and 1:10 (donated as R1 to R5). Start (the operation start-up, the 0 h) and End (the operation end, the 48th h).

**Table 3:** The function and abundance of the bacteria in genus in reactors at different stages.

Genus	Function	References
Chlorella	Absorbing inorganic carbon, nutrient removal	^52^
Scenedesmus	Absorbing inorganic carbon, nutrient removal	^53^
Anabaena	Absorbing inorganic carbon, nutrient removal	^47^
Oscillatoria	Absorbing inorganic carbon, nutrient removal	^30^
Acinetobacter	COD removal	^31^
Unclassified Pseudomonadales	Heterotrophic nitrification, phosphorus removal	^54^
Unclassified Proteobacteria	COD removal	^29^
Unclassified Bacteroidetes	Anaerobic reaction	^15^
Burkholderiales	Ammoxidation	^29^
Unclassified Betaproteobacteria	Ammoxidation	^29^
Pseudomonas	Heterotrophic nitrification, denitrification	^54^
Comamonas	Ammoxidation	^54^
Nitrospira	Nitration	^33^
Unclassified_p_Chloroflexi	Photosynthesis, nutrient removal	^32^
Unclassified_c_Gammaproteobacteria	COD removal	^55^
Rhodobacterales	Denitrification	^56^
Unclassified_o_Myxococcales	Nitrogen removal	^29^
Unclassified_c_Alphaproteobacteria	Anaerobic oxidation	^29^
Thauera	Heterotrophic nitrification, Phosphorus removal	^57^

#### Microbial community structure at End

At End, the community related to nutrient removal declined in bacteria community with all consortia. For example, the bacteria community related to denitrification process (Table S1), such as *Pseudomonas*, changed to weak community, down to 0.22%, 1.92%, 0.49%, 0.40% and 0.67%, respectively (Table S1). However, the ratio of algae community related to photosynthesis and bacteria community about heterotroph was 10.10:1, 6.58:1, 6.32:1, 6.06:1 and 5.05:1 respectively (Table 2).

#### Convergence of community succession

All consortia with R1 to R5 at End was similarity to each other by Bray–Curtis similarity, which the succession of community from R1 to R5 has convergence. The Bray–Curtis similarity in R2 was smallest than other consortia, followed by R1, R3, R4 and R5 (0.88, 0.89, 0.58, 0.25 and 0.18 respectively). The highest nitrogen, phosphorus removal efficiencies and biomassproductivity were observed with 5:1 algae and sludge consortium (73.7%, 94.3% and, 98.2% respectively) within 48 h, which was higher and faster than those with other ratios. The proportion of autotrophic microorganism community and heterotrophic microorganism, 86.815% and 13.185% at End respectively (Table S1), got tiny changed with the process of wastewater treatment which was named *SDJZ-CC1*.

## Discussion

### Introduction of macroecology theory into phycosphere

The theories of phycosphere space–time evolution was only guided by the judgement of microbiologist Beijerinck that “environmental conditions determine microbes”^34^. In fact, phycosphere is a complete ecosystem^35^. Inorganic components including matter and energy, while producers, consumers and decomposers are composed of microbes^36^. Therefore, the macroecology should be introduced into revealing the mechanism of algae-bacterial consortium in phycosphere. At first, the community succession refers to the adjustment of the community structure and relationship between algae and bacteria under the influence of ecological factors such as nutrition, DO, pH, temperature and light^37^. The result of community succession is the Climax Community in which the most complex community structure and the most diverse community relationships exist under the specific nutrient conditions in wastewater^19^. The efficient use of nutrients is maximized in climax community in phycosphere. There is an optimum treatment effect to remove C, N and P for the algal–bacterial consortium in climax community. Therefore, the component with the best treatment effect in wastewater existed the climax community in secondary effluent. R2 gradually succeeded to the climax community in secondary effluent treatment process.

Producers (autotrophs) are composed of microalgae or autotrophs^38^. Producers, such as algae, cyanobacteria or photosynthetic bacteria, can absorb the light energy and inorganic carbon in wastewater and convert it into organic matter to fix into the algal- bacterial consortia by photosynthesis^39^. Inorganic nitrogen is brought into organic nitrogen by nitrifying bacteria, such as *Nitrospira.* Producers convert inorganic carbon or inorganic nitrogen into organic forms in the phycosphere. Organizers participate in material circulation and signal transmission of the consortium. For example, the microalgae fix the inorganic carbon into organic carbon that is oxidized by bacteria to get energy. The CO_2_ produced by bacterial oxidation is carbon source for photosynthesis of algae. The Decomposer can convert organic matter into inorganic matter. For example, *Acinetobacter*, the aerobic bacteria which can remove COD, oxidizes the organic carbon source into CO_2_ to the growth of algae.

The community convergence is a product of community succession in the phycosphere. It is universally accepted that resources determine the direction of community convergence and there is only one climax community where resource and environmental conditions are established. Dominant populations dominate resources of the ecosystem. The number of competitors is limited and co-living may promote each other’s growth. Community succession needs to be proven to be utilized by Community similarity analysis which needs statistical tools. Bray–Curtis similarity is often used by ecologists to quantify differences between samples based on abundance or counting data. In essence, it refers to a quantitative value, the size of which reflects the difference in community species composition between different samples within each group^20^. Bray–Curtis similarity clustered similar DNA sequences into a smaller number of taxa to improve the efficiency of analysis and analytical accuracy^24^.

### Driving force of community succession

Nutrients (resource) drive the direction of succession of communities^39,40^. The level of nutrients determines the end point of the development of the ecological community^19^. And the ecological factors determine the development process of ecological community^39^. In this study, great utilization of nitrogen and phosphorus and aerobic environment caused by the photosynthesis and aeration led to autotrophic succession^26^. Therefore, convergence of succession occurs commonly in all consortia in the study (Table 4, Fig. 3). The efficiency of resource utilization by the community is consistent with the removal efficiency of nitrogen and phosphorus in wastewater^40^. In other words, the climax community was the destination of the algal–bacterial consortium whose nitrogen and phosphorus removal were fastest and highest with all consortia. Therefore, the initial concentration of R2 would eventually develop into the climax community (Table 4). In aquatic ecosystem, the similarity tendency got reappearance^41^. For example, Cruz and Pompeu^41^ revealed The importance of variables linking habitat diversity and basin connectivity to fish assemblage richness and structure by Bray–Curtis coefficients. And the similarity of fish assemblage community structure changes with time and space was also studied. In addition, there are similar studies in microecology and the distribution of the functional genes in suspended activated sludge system, such as antibiotic resistance genes^22,24^, was tested by Bray–Curtis coefficients.

**Table 4:** Bray–Curtis Similarity index of R1 to R5 at Stage A and Stage B.

		Start					End				
		R1	R2	R3	R4	R5	R1	R2	R3	R4	R5
Start	R1	1	0.92	0.60	0.26	0.19	0.91	0.88	0.91	0.90	0.88
	R2	0.92	1	0.67	0.33	0.27	0.88	0.89	0.91	0.92	0.92
	R3	0.60	0.67	1	0.66	0.59	0.55	0.58	0.61	0.63	0.66
	R4	0.26	0.33	0.66	1	0.93	0.21	0.25	0.27	0.29	0.32
	R5	0.19	0.27	0.59	0.93	1	0.15	0.18	0.21	0.22	0.25
End	R1	0.91	0.88	0.55	0.21	0.15	1	0.93	0.94	0.90	0.89
	R2	0.89	0.89	0.58	0.25	0.18	0.93	1	0.94	0.94	0.91
	R3	0.91	0.91	0.61	0.27	0.21	0.94	0.94	1	0.95	0.95
	R4	0.90	0.92	0.63	0.29	0.22	0.90	0.94	0.95	1	0.95
	R5	0.88	0.92	0.66	0.32	0.25	0.89	0.91	0.95	0.95	1

R = 0.452, p = 0.0083. Consortia with algae/sludge 10:1, 5:1, 1:1, 1:5 and 1:10 (donated as R1 to R5). Start (the operation start-up, the 0 h) and End (the operation end, the 48th h).

Convergence and climax community can deduce the optimal proportion of algae and bacteria in the initial community. As shown in Table 4, the Bray–Curtis similarity index of R2 at Start was closer to each component of End, especially the high proportion of sludge, such as R4 and R5. It was indicated that the community was succeeding in the direction closer to R2. However, R2 developed to the Climax Community faster when using environmental resources. Because R2 at Start had better ability of assimilation of nitrogen and phosphorus and coordinated symbiosis between biological communities. This result can be used to select appropriate algae and bacterial consortia to optimize wastewater removal efficiency and improve the biomass production capacity. The *SDJZ-CC1* exists in an aerobic environment and its dominant species are autotrophs, such as microalgae community. This community structure leads to high nitrogen and phosphorus assimilation efficiency and biomass growth capacity.

### Effects of community structure on wastewater treatment process

The autotrophs or heterotrophic microorganisms with the largest number in the climax community are called the dominant species^42^. The dominant species play an important role in the removal of nutrients^42^. In autotrophic microorganism dominant species, the removal of TDP was independent of the bacteria community since phosphorus accumulating bacteria can become dominant species in the alternate of aerobic and anaerobic process (Table 3)^25^. In addition, P removal was relevant to the assimilation by algae in aerobic autotrophic environment. Besides, in high pH values environment, which caused by photosynthesis, P can chemically be precipitated and be removed in wastewater. Therefore, there was little direct effect for bacteria community on TDP removal^43^. And the effect of removal TDP of consortium is relevant with a high proportion of algae^43^. However, excess algae can obscure the light-exposed area in the reactor, which impedes the ability of algae to absorb TDP^10^. The low proportion of bacteria inhibited the symbiosis between algae and bacteria^10^. It is generally believed that the symbiotic relationship between alga and bacteria promotes TDP absorption by algae^9^. The similarity is that nitrogen was removed by an algal–bacterial consortium. In this study, nitrogen removal is through assimilation of microalgae communities, because denitrification must belong to anaerobic bacteria in facultative anaerobic environment, rather than under aerobic condition (aeration or photosynthesis)^26^. In addition, the increasing of pH, due to the photosynthesis, affected the NH_3_-N removal. With pH value increasing, the free ammonia can be formed and volatilized. Therefore, nitrogen removal is related to the growth of microalgae^44^. The growth of microalgae is related to nitrogen absorption. Removal of NH_3_-N is preferred over TDN due to nitrification by ammonia-oxidizing bacteria, converting NH_3_-N to nitrate nitrogen which is good for absorption^45^. The growth of microalgae community increased the ability to absorb nutrition. The microalgae community was the dominant Species which gives priority to nutrition (Fig. 4). Therefore, competitive relations which occur between microcystis and alginolytic bacteria^46^ may arise between microalgae communities and nitrifying bacteria, such as *Pseudomonadales*, *Nitrospira* (Fig. 4). The ratio of *Chlorella* and *Scenedesmus* increased due to the better absorptive capacity of *Chlorella* than *Scenedesmus* to produce bio-oil (Fig. 4)*.* The similarity lies in the growth of *Anabaena*^47^ and *Oscillatoria*^31^, belonging to cyanobacteria, decreased due to the competition among the microalgae community. In connection with previous literature, Liang et al.^48^ achieved higher removal efficiencies of NH_3_-N (86.0%) and TDP (93.0%). In a similar wastewater environment (about NH_3_-N 20 and TDP 4 mg L^−1^ in the initial), the results obtained from the present study indicated the higher removal efficiencies of NH_3_-N ($>$ 85.0% and the study 93.26%) and TDP ($>$ 90.0% and the study 94.4%).

As shown in Table 3, the dominant Species, in heterotrophic microorganism species, is *Proteobacteria_unclassified* and other bacteria belonging to *Proteobacteria* which has the ability to remove organic matter, nitrogen and phosphorus. However, the ability of *Proteobacteria* to remove nitrogen and phosphorus was not significant because the dominant Species of nitrogen and phosphorus was *Chlorella.* Therefore, the ability of *Proteobacteria,* such as α-*Proteobacteria, β-Proteobacteria, and γ-Proteobacteria,* to remove organic matter was significant^29^. The CO_2_ and low molecular weight organic matter produced by *Proteobacteria* oxidation are provided to microalgae community. Therefore, COD is mainly oxidized by the bacterial community rather than microalgae community. And the growth of the community structure of the bacterial community about nitration, denitrification and release of phosphorus was decreased.

It is generally acknowledged that bacterial communities prefer acidic environments while microalgae communities thrive in alkaline environments. The increase of pH directly led to the change of community structure straightly (Fig. 3). Generally, the pH of the water environment where consortia of algae and bacteria live increases with the decrease of CO_2_ concentration. The reason why the ratio 5:1 of the consortium was the highest is that symbiosis enhances the ability of the consortium to absorb CO_2_^49^. Similarly, Su et al.^10^ conducted a similar study on the highest nitrogen and phosphorus removal efficiencies observed with 5:1 for ratio of algae and sludge consortium, 91% and 93.5% respectively.

### Effects on biomass production

The growth of biomass is related to carbon source and nutrition recovery^8^. Biomassproductivity and quality determine that the cost can be offset in the commercial application of wastewater treatment based on microalgae wastewater treatment^8^. And production of biomass is the most direct expression of consortium’s resource utilization^50^. The biomass productivity of R2 was the largest among all consortia (Fig. 2). The removal of nutrients is also the most efficient (Fig. 3). Microalgae biomass was gradually accumulated, due to nitrogen supply shifted from N-rich to N-deficient condition was the absorption of algae with aeration oxygenation^26^. Therefore, the removal of nitrogen and the generation of algae biomass can be carried out simultaneously. However, the removal of TDP precedes the growth of biomass, because there may be a buffer period for the assimilation of TDP by algae^43^. In connection with previous literature, the results of this study show that the biomass production was related to the concentration of algae, with a high ratio of algae/sludge at consortium^51^. The balance of victory was in favor of microalgae community, due to the dominance of their dominant community. In the microalgae community, *Chlorella* is considered to be a kind of quality algae to produce oils^25^.

### Novel insight in consortium for wastewater treatment and community structure

This study first explored that theory of ecology was introduced into phycosphere. Convergence of community succession in algal–bacterial consortia was demonstrated by Bray–Curtis similarity. The result of nutrients removal in consortia was consistent with the resource utilization in macroecology. In community structure, the algae, the role of productors, showed a high potential for biomass production by utilizing various inorganic carbon substrates on photosynthesis in aerobic autotrophic environment. Besides, bacteria, the role of consumers or decomposers, oxidized organic matter to promote the inorganic carbon substrates. Above all suggest that macroecology perspectives can explain community structure in phycosphere. Therefore, convergence of community succession in algal–bacterial consortia were explained based on macroecology. This is of great significance for exploring the shortening of community culture time and predicting ecological community.

## Conclusions

In this study, the ‘convergence’ of community structure in the algal–bacterial consortia with different ratios of algae/sludge was analyzed confirmed by metagenomic sequencing and Bray–Curtis similarity, and ‘convergence’ which is well known as in macroecology was the succession results of community succession in macroecology the algal–bacterial consortia. It means that stable and similarity climax communities have eventually established in wastewater, no matter what the ratio of algae/sludge was in the initial. The consortium with 5:1 ratio of algae/sludge, which community structure was most similar to that at end, namely the climax community. The consortium, and achieved the highest nitrogen and phosphorus removal efficiency and biomass production. For this phenomenon ecology, it's the best ecological explanation that the highest resource utilization (nutrient contaminant uptake) climax community can be accessed. Therefore, the novel insight based on authenticated the theory well-established in macroecology introduced into the phycosphere can be employed to optimize their community structure to enhance shorten culture time of consortium for advanced wastewater treatment.

## Supplementary Information

Supplementary Information.
