# Supplementary material for: Convergent community structure of algal–bacterial consortia and its effects on advanced wastewater treatment and biomass production
Source: Sci Rep. 2021 Oct 26;11:21118. doi: 10.1038/s41598-021-00517-x (PMC8548336; doi:10.1038/s41598-021-00517-x)
Supplement: Supplementary file 1 — Supplementary Information. [file 41598_2021_517_MOESM1_ESM.pdf]

Supplementary Information

Supplementary Table S1 The abundance of Community in genus in consortia at different stages

Community	Abundance									
	Stage A					Stage B				
	R1	R2	R3	R4	R5	R1	R2	R3	R4	R5
Chlorella	81.537%	75.167%	45.427%	15.033%	9.034%	77.473%	73.449%	72.996%	71.593%	69.572%
Scenedesmus	9.363%	8.167%	5.273%	1.633%	0.966%	12.912%	12.720%	11.967%	11.932%	11.595%
Anabaena	0.780%	1.552%	4.427%	8.141%	8.614%	0.339%	0.326%	0.698%	1.307%	1.307%
Spirulina	0.780%	1.397%	4.471%	8.036%	8.687%	0.268%	0.320%	0.687%	1.000%	1.000%
Acinetobacter	0.010%	0.020%	0.067%	0.098%	0.149%	1.674%	2.996%	1.640%	4.059%	1.751%
Unclassified_o_Pseudomonadales	0.004%	0.007%	0.022%	0.035%	0.039%	3.323%	0.143%	3.074%	0.478%	1.746%
Unclassified_p_Proteobacteria	0.790%	1.451%	4.268%	7.288%	7.768%	0.756%	0.847%	0.912%	1.020%	1.361%
Unclassified_p_Bacteroidetes	0.658%	1.309%	3.771%	6.491%	7.039%	0.212%	0.322%	0.958%	1.018%	1.231%
Burkholderiales	0.513%	0.930%	2.566%	4.073%	4.613%	0.471%	0.878%	0.927%	0.651%	1.397%
Unclassified_c_Betaproteobacteria	0.509%	0.952%	2.806%	4.855%	5.191%	0.102%	0.301%	0.492%	0.562%	0.885%
Pseudomonas	0.078%	0.161%	0.438%	0.739%	0.788%	0.131%	1.832%	0.167%	0.113%	0.344%
Comamonas	0.006%	0.048%	0.036%	0.058%	0.073%	0.023%	1.802%	0.042%	0.064%	0.174%
Nitrospira	0.254%	0.657%	2.377%	4.106%	4.195%	0.008%	0.018%	0.072%	0.193%	0.169%
Unclassified_p_Chloroflexi	0.211%	0.489%	1.459%	2.830%	3.108%	0.020%	0.046%	0.147%	0.416%	0.336%
Unclassified_c_Gammaproteobacteria	0.153%	0.273%	0.786%	1.353%	1.445%	0.151%	0.242%	0.222%	0.313%	0.295%
Rhodobacterales	0.219%	0.306%	0.763%	0.882%	1.118%	0.084%	0.084%	0.321%	0.289%	0.329%
Unclassified_o_Myxococcales	0.218%	0.383%	1.151%	2.031%	2.266%	0.016%	0.041%	0.131%	0.244%	0.247%
Unclassified_c_Alphaproteobacteria	0.196%	0.297%	0.799%	1.076%	1.257%	0.096%	0.076%	0.218%	0.263%	0.266%
Thauera	0.107%	0.197%	0.584%	1.010%	1.101%	0.084%	0.158%	0.317%	0.106%	0.467%

Consortia with algae/sludge 10:1, 5:1, 1:1, 1:5 and 1:10 (donated as R1 to R5). Start (the operation start-up, the 0 hour) and End (the operation end, the 48th hour)
